# Suppression of Transmembrane Tumor Necrosis Factor Alpha Processing by a Specific Antibody Protects Against Colitis-Associated Cancer

**DOI:** 10.3389/fimmu.2021.687874

**Published:** 2021-10-05

**Authors:** Hongping Ba, Rui Jiang, Meng Zhang, Bingjiao Yin, Jing Wang, Zhuoya Li, Baihua Li, Xiaoxi Zhou

**Affiliations:** ^1^ Department of Immunology, Tongji Medical College, Huazhong University of Science and Technology, Wuhan, China; ^2^ Department of Hematology, Tongji Hospital, Huazhong University of Science and Technology, Wuhan, China

**Keywords:** transmembrane tumor necrosis factor-α, soluble tumor necrosis factor-α, colitis-associated cancer, antibody-based therapy, cytokines, macrophages, regulatory T cells, myeloid-derived suppressor cells

## Abstract

Soluble tumor necrosis factor-α (sTNF-α) plays an important role in colitis-associated cancer (CAC); however, little is known about transmembrane TNF-α (tmTNF-α). Here, we observed an increase in sTNF-α mainly in colitis tissues from an azoxymethane/dextran sodium sulfate (DSS)-induced CAC mouse model whereas tmTNF-α levels were chiefly increased on epithelial cells at the tumor stage. The ratio of intracolonic tmTNF-α/sTNF-α was negatively correlated with the levels of pro-inflammatory mediators (IL-1β, IL-6, and NO) and M1 macrophages but positively correlated with the infiltration of myeloid-derived suppressor cells, regulatory T cells, and the level of the anti-inflammatory cytokine IL-10, suggesting an anti-inflammatory effect of tmTNF-α. This effect of tmTNF-α was confirmed again by the induction of resistance to LPS in colonic epithelial cell lines NCM460 and HCoEpiC through the addition of exogenous tmTNF-α or transfection of the tmTNF-α leading sequence that lacks the extracellular segment but retains the intracellular domain of tmTNF-α. A tmTNF-α antibody was used to block tmTNF-α shedding after the first or second round of inflammation induction by DSS drinking to shift the time window of tmTNF-α expression ahead to the inflammation stage. Antibody treatment significantly alleviated inflammation and suppressed subsequent adenoma formation, accompanied by increased apoptosis. An antitumor effect was also observed when the antibody was administered at the malignant phase of CAC. Our results reveal tmTNF-α as a novel molecular marker for malignant transformation in CAC and provide a new insight into blocking the pathological process by targeting tmTNF-α processing.

## Introduction

Up to 20% of all human cancers result from chronic inflammation and persistent infection. Patients who suffer from inflammatory bowel disease (IBD) have a high risk of developing colitis-associated colorectal cancer (CAC) and have a high mortality from the disease (1, 2). Chronic inflammation contributes to the development of low- and high-grade dysplasia that further converts colitis into colorectal cancer (CRC). Proinflammatory cytokines and tumor-infiltrating myeloid and immune cells play critical roles in the initiation, promotion, and progression to malignant transformation (3–8).

Tumor necrosis factor-α (TNF-α) exists in two bioactive forms: 26-kDa transmembrane TNF-α (tmTNF-α) and 17-kDa soluble TNF-α (sTNF-α). tmTNF-α on the cell surface is cleaved by a metalloproteinase, TNF-α-converting enzyme (TACE), to release sTNF-α. Both forms of TNF-α are bioactive and display distinct functions. sTNF-α, a proinflammatory factor, plays a pivotal role in the pathogenesis of IBD. The expression of TNF-α is elevated in biopsies and peripheral blood cells (PBC) obtained from patients with ulcerative colitis (UC) and Crohn’s disease (CD) (9, 10). Serum levels of sTNF-α are markedly increased by 1.7-fold in patients with active UC (11), which contributes to the mucosal damage and chronic inflammation responsible for the signs and symptoms of active UC. The release of sTNF-α also increases in the mouse colon following azoxymethane/dextran sodium sulfate (AOM/DSS) treatment, and ablation of TNF receptor (TNFR) 1 results in reduced mucosal damage, macrophage and neutrophil recruitment, and tumor formation in mouse colon, suggesting a tumor-promoting role of TNF-α in CAC (12). In addition, TNF-α also promotes CRC metastasis. The efficacy of blocking TNF-α signaling by anti-TNF-α agents has been reported in the treatment of IBD (13, 14). TNF-α antagonists not only inhibit CAC induction in mice by limiting TNF-induced infiltration of neutrophils and macrophages (12) but also reduce the risk of both dysplasia and CAC when combined with other anti-inflammatory medications in the clinic (15).

The role of tmTNF-α in IBD has been clarified in studies on the mechanisms of anti-TNF-α agents. Although the affinity of anti-TNF-α agents for tmTNF-α is lower than that for sTNF-α, the binding and neutralization of tmTNF-α by these agents is presumed to be crucial for their different clinical efficacies, as the neutralization of sTNF-α alone or transferring T cells expressing a non-cleavable tmTNF-α mutant that does not produce sTNF-α did not protect the mice from intestinal inflammation (16, 17). However, a deficiency in both IL-10 and TNF-α exacerbates enterocolitis in mice, indicating some protective effects of TNF-α on this condition (18). Selective inhibition of sTNF-α by XPro1595-DN-TNF significantly prevents chemical-induced carcinogenesis (19), indicating a possible protective effect of tmTNF-α. In addition, tmTNF-α functions not only as a ligand that binds TNFRs to induce forward signaling but also as a receptor to transduce outside-to-inside signals, namely, reverse signaling. The mechanisms underlying the benefit of anti-TNF agents in patients with IBD are not limited to the neutralization of both forms of TNF-α, as these agents activate reverse signaling from tmTNF-α. Binding of infliximab to tmTNF-α not only induces apoptosis but also downregulates proinflammatory mediators and upregulates the anti-inflammatory cytokines IL-10 and TGF-β (20–23), indicating that tmTNF-α-mediated reverse signaling promotes the resolution of inflammation. However, little is known about tmTNF-α expression and its function during the development of CAC. Here, we found that sTNF-α levels increased in the inflammation phase, while tmTNF-α expression was enhanced in colon tissues during malignant transformation in AOM/DSS-induced CAC mice. The administration of a tmTNF-α antibody to shift the time window of tmTNF-α expression ahead to the inflammation phase significantly suppressed inflammation and limited subsequent tumor formation.

## Materials and Methods

### Mouse Model

All animal experiments were approved by the Animal Care and Use Committee of Huazhong University of Science and Technology. Male C57BL/6 mice, 4 to 6 weeks old, were purchased from Beijing HFK Bioscience Company (Beijing, China) and housed under specific pathogen-free conditions with free access to food and water. Mice were intraperitoneally injected with 10 mg/kg AOM (Sigma-Aldrich, St. Louis, MO, USA) on day -7, followed by three 5 day cycles of administration of 2.5% DSS (MP Biomedicals, Santa Ana, CA) in the drinking water with a 14 day intercycle interval starting 1 week after the AOM injection (day 0). Mice were sacrificed 13 days after the end of the last cycle. Mice were weighed every three days. Colon tissues were dissected from the mice, flushed and cleaned with PBS, and cut open longitudinally to examine tumor nodules. The tumor diameter was measured with Vernier calipers.

### Disease Activity Index (DAI)

Body weight, stool consistency, and occult or gross blood were analyzed every three days. The disease activity index score was assessed in a blind manner as follows (24): (1) Body weight loss: 0: none; 1: 1–5%; 2: 6–10%; 3: 11–20%; 4: > 20%; (2) Stool consistency: 0: normal; 2: loose stool; 3 and 4: diarrhea (adhering to the anus); and (3) Hematochezia: 0: negative; 2: positive hemoccult; and 4: gross bleeding.

The hemoccult test was performed using a solution composed of 1% o-tolidine in 80 ml of glacial acetic acid, 20 ml of absolute ethanol, and 3% hydrogen peroxide (25).

### Cell Culture, Transfection, and Stimulation

Two human colonic cell lines - NCM460, a gift from Prof. Junbo Hu (Department of Gastrointestinal Surgery Center, Tongji Hospital, Tongji Medical College, Huazhong University of Science and Technology, Wuhan, China) and HCoEpiC (Otwo Biotech, Shenzhen, China) were cultured at 37°C in a 5% CO_2_ atmosphere with DMEM medium (Life Technologies, USA) supplemented with 10% heat-inactivated, pyrogen-free fetal calf serum (FCS, Sijiqing, Hangzhou, China), 1 mM sodium pyruvate, 2 mM L-glutamine, 100 U/ml penicillin, and 100 mg/ml streptomycin.

The full-length human TNF-α cDNA and its leader sequence (LS) mutant were generated by PCR from the pCDNA 3.0 plasmid containing TNF-α or TNF-LS (26) and cloned into the pHAGE-CMV-MCS-PGK puro vector at the Bam HI and Xho I sites. The primers were synthesized by TSINGKE Biological Technology (Beijing, China), and their sequences are listed in **Supplementary Table 1**. The two constructs were verified by DNA sequencing (TSINGKE Biological Technology, Beijing, China). Recombinant lentiviruses were produced by transient four-plasmid cotransfection into 293T cells and purified by ultracentrifugation (27). NCM460 cells were transfected with the lentivirus in antibiotic-free growth medium containing 2 µg/ml polybrene (Sigma-Aldrich, St. Louis, MO, USA) and incubated overnight. Cells were selected with 2 µg/ml puromycin for 2 weeks and subcloned using the limiting dilution method. The expression of TNF-α and TNF-LS on the cell surface was monitored for positive clone selection. HCoEpiC cells were transiently transfected with pCDNA 3.0 plasmid containing TNF-α or TNF-LS using polyethylenimine Max, Linear, MW 40,000 (Polysciences Inc., Illinois, USA) for 48 h.

For the detection of tmTNF-α−mediated forward signaling, 293T cells stably transfected with human tmTNF-α were fixed with 4% paraformaldehyde for 30 min at room temperature (RT) and used as the source of exogenous tmTNF-α (28). 100 ng/ml sTNF-α (Peprotech, Rocky Hill, NJ) or tmTNF-α-overexpressing 293T cells as effector cells were cocultured with NCM460 or HCoEpiC cells as target cells at an effector/target (E/T) ratio of 10:1 in the presence of 10 ng/ml LPS (from *Escherichia* coli 026:B6, no. L2654, Sigma-Aldrich, St. Louis, MO, USA) for different times.

### Preparation of the Single-Cell Suspension and Flow Cytometry

Cell suspensions from mouse spleen were prepared as previously described (29). Cell suspensions were prepared from mesenteric lymph nodes (MLNs) by mechanically disrupting MLNs, and colonic epithelial cell suspensions were prepared by digesting colon tissues with 1 mM EDTA and 1 mm dithiothreitol (30). Single-cell suspensions were obtained by filtering the aforementioned cell suspensions through a 75 μm 200 mesh filter (24).

Splenic or MLN cells were stained with the following fluorescent dye-conjugated antibodies (eBioscience, San Diego, CA, USA) for 30 min at 4°C: APC-F4/80 (Cat# 17-4801), PE-CD11b (Cat# 12-0122), FITC-Gr1 (Cat# 11-6041), FITC-CD4 (Cat# 11-0041), APC-IL-17 (Cat# 17-7177), APC-CD25 (Cat# 17-0251) and PE-Foxp3 (Cat# 12-4771). For the analysis of tmTNF-α expression, NCM460 cells, HCoEpiC cells or colonic epithelial cells were stained with a monoclonal antibody against tmTNF-α (31) for 30 min at 4°C, followed by a FITC-conjugated secondary antibody (FeiYi, Wuhan, China, Cat# ZF-0312). The stained cells were analyzed using an LSRII flow cytometer (Becton Dickinson, San Jose, CA, USA).

### Culture of Colonic Tissue for the Detection of Both Forms of TNF-α

Fifty milligrams of tissue from the distal portion of the colon were washed with 1x PBS and then cut into segments of ≈ 1 cm^2^. Colonic tissue samples were cultured in RPMI 1640 containing 5% fetal bovine serum in a 24-well culture plate for 24 h (24). Supernatants were collected for the detection of sTNF-α; and membrane proteins were extracted according to the manufacturer’s protocol (Biovision, Milpitas, CA, USA) for the detection of tmTNF-α. Concentrations of both forms of TNF-α in colonic tissue or serum sTNF-α were detected using a TNF-α ELISA kit (eBioscience, San Diego, CA, USA). The ratio of tmTNF-α and sTNF-α in colonic tissue was calculated.

### Detection of Cytokines and Nitric Oxide (NO)

For the preparation of tissue homogenates, distal colons (2 cm in length; 1 cm away from the anus) were cut and flushed with 1x PBS to remove gut contents and homogenized in RIPA buffer (Beyotime Biotechnology, Shanghai, China), followed by centrifugation at 12000 rpm for 20 min.

The concentrations of IL-1β, IL-6, IL-10, and TGF-β in colonic homogenates or in supernatants of cultured cells were detected using ELISAs (eBioscience, San Diego, CA, USA) according to the manufacturer’s instructions. NO was quantified using a spectrophotometric assay based on the Griess reaction with a commercial NO assay kit (Beyotime Biotechnology, Shanghai, China).

### Western Blot Analysis

Total protein was extracted by lysing cells in lysis buffer (20 mM HEPES, pH 7.4, 20 mM NaCl, 10% glycerol, and 1% Triton X-100). Colonic membrane proteins were prepared using a Membrane Protein Extraction Kit (Biovision, Milpitas, CA, USA) and soluble proteins were isolated from colon homogenates using methanol and chloroform (28). All protein samples were subjected to 12% SDS-polyacrylamide gel electrophoresis and transferred to PVDF membranes (Millipore, Merck KGaA, Darmstadt, Germany). Immunoblotting was performed with the following primary antibodies: anti-tmTNF-α (home-made) (31), anti-TNF-α (Cat# 3707s), anti-PARP (Cat# 9532s), anti-cleaved caspase 3 (Asp175) (Cat# 9661s) from Cell Signaling Technology (Danvers, MA, USA), anti-IκB-α (Santa Cruz, CA, USA, Cat# sc-1643), anti-p65 (Cat# A19653), anti-p-p65 (Cat# AP0475), anti-caspase 3 (Cat# A17900), anti-Na^+^/K^+^ ATPase (Cat# A12405), and anti-β-actin (Cat# AC026) from Abclonal (Wuhan, China). HRP-conjugated secondary antibodies (Cell Signaling Technology, Danvers, MA, USA, Cat# 7074) were subsequently applied to the membrane. Bands were visualized using an enhanced chemiluminescence system (ECL; TIANGEN, Beijing, China).

### Real-Time PCR

Total RNA was extracted from NCM460 cells using TRIzol reagent (Invitrogen, USA). The cDNA templates were reverse transcribed from 1 μg of RNA with a HiFiScript cDNA Synthesis Kit (Yeasen, Shanghai, China) according to the manufacturer’s instructions. Relative mRNA levels of *IL-6* and *iNOS* were determined using real-time PCR with UltraSYBR Mixture (Yeasen, Shanghai, China). PCR was performed using the following conditions: 95°C for 10 min, followed by 40 cycles of 95°C for 15 s and 60°C for 1 min. The results were analyzed using the 2^−△△Ct^ method and normalized to the corresponding levels of GAPDH. The primers were synthesized by Sangon Biotech (Shanghai, China) and are listed in **Supplementary Table 1**.

### Histopathology and Immunohistochemistry

Colonic tissue sections (4 μm) were deparaffinized, rehydrated, and stained with hematoxylin and eosin (H&E). Colonic tissue sections (4 μm) were dewaxed with xylene and rehydrated in graded ethanol solutions. Antigen retrieval was performed on the sections using Antigen Unmasking Solution (Boster Biological Technology, Wuhan, China). Immunohistochemical staining was performed using the avidin–biotin complex method with anti-mouse TNF-α (Boster Biological Technology, Wuhan, China, Cat# BA0131), anti-mouse F4/80 (eBioscience, San Diego, CA, USA, Cat# 14-4801), anti-mouse CD16/32 and anti-mouse IL-17 (BD Biosciences, San Jose, CA, USA, Cat# 553141 and Cat# 559501), anti-mouse CD206 (AbD Serotec, Kidlington, UK, Cat# MCA2235GA), anti-mouse Foxp3 (Cat# 12653s), and anti-mouse Gr1 monoclonal antibodies (Cat# 31469s) from Cell Signaling Technology (Danvers, MA, USA), followed by HRP-conjugated universal anti-mouse IgG/anti-rabbit IgG antibody (Boster Biological Technology, Wuhan, China, Cat# SA1052/Cat# SA1055). Signals were visualized using an ImmPACT DAB peroxidase substrate (Boster Biological Technology, Wuhan, China), followed by counterstaining with hematoxylin (Sigma Aldrich, St. Louis, MO). Images were viewed and captured with an Olympus BH-2 light microscope (Olympus, Tokyo, Japan) attached to a computerized imaging system. The positive cells in five randomly selected fields were counted at 200× or 400× magnification with Image-Pro Plus Version 6.0 software (Media Cybernetics, Bethesda, MD, USA).

### Apoptosis Detection

*In situ* TUNEL staining was performed using an *In Situ* Cell Death Detection Kit (Roche Diagnostics, Basel, Switzerland) according to the manufacturer’s instructions. In brief, sections of colonic tissue were deparaffinized, repaired with proteinase K at 37°C for 25 min, and permeabilized at RT for 20 min. Sections were incubated with TdT and fluorescent dye-labeled dUTP for 1 h at 37°C, followed by DAPI staining at RT for 10 min. Apoptotic cells were observed using a laser scanning confocal microscope (Nikon D-Eclipse CI, Tokyo, Japan).

### Statistical Analysis

Student’s t-test and one-way or two-way analysis of variance followed by Tukey’s *post hoc* test were used to compare data from two or multiple groups with GraphPad Prism 6.0 software (San Diego, CA, USA). *P* values less than 0.05 were considered statistically significant.

## Results

### sTNF-α Levels Increase in the Inflammatory Phase, While tmTNF-α Levels Are Elevated in the Tumor Phase of the Mouse AOM/DSS-Induced CAC Model

**Figure 1:**
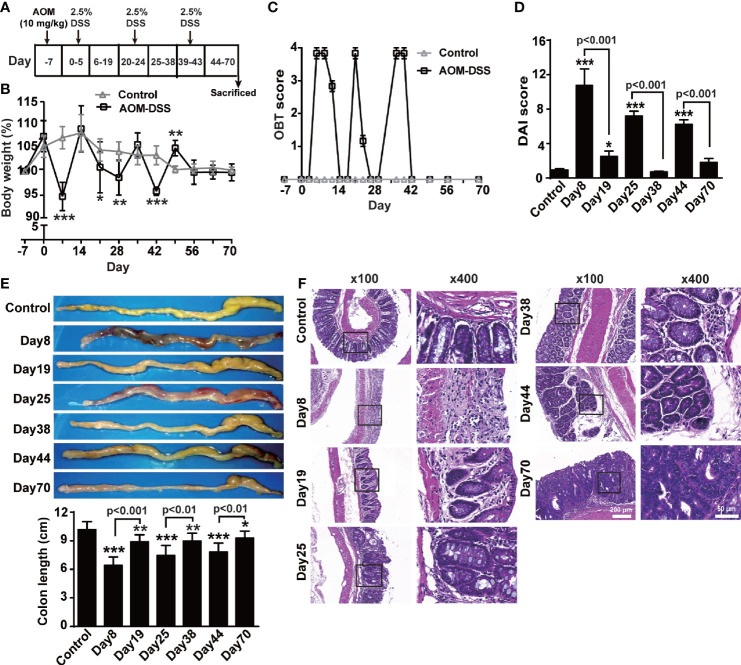
AOM/DSS induced CAC. **(A)** Schematic treatment of mice with AOM and DSS. Mice were intraperitoneally administered with AOM (10 mg/Kg) at day -7, followed by three cycles of drinking 2.5% DSS-containing water for 5 days with interval of 14 days (n = 6, each group). Time course for body weight **(B)**, score of occult blood test (OBT) **(C)** and disease activity index (DAI) **(D)**. **(E)** Representative images and quantitative data of colon length. **(F)** Colons tissue sections were stained with hematoxylin and eosin. Representative histopathological images (×100, ×400). All quantitative data are expressed as means ± SEM. *P < 0.05, **P < 0.01, ***P < 0.001 versus control.

Mice were intraperitoneally injected with AOM (10 mg/kg) on day -7, followed by three cycles of drinking 2.5% DSS-containing water for 5 days with an interval of 14 days (**Figure 1A**) to determine the dynamical changes in the levels of both forms of TNF-α during the transformation from colitis to tumors. After the first round of drinking DSS-containing water (day 8), the mice developed acute colitis, showing diarrhea, weight loss (**Figure 1B**), fecal occult blood (**Figure 1C**) and even rectal bleeding, an increased DAI score (**Figure 1D**), and an obvious hyperemic, shortened colon filled with bloody stools (**Figure 1E**). The histopathological changes included the infiltration of a large number of inflammatory cells into the lamina propria of the colon and significant damage to the intestinal epithelial barrier (**Figure 1F**). These symptoms and pathological changes were markedly alleviated after a two-week interval (day 19), and the body weight and intestinal structure were close to normal. After the second round of DSS administration (day 25), repeated inflammation was induced, inflammatory cells infiltrated the submucosal layer, and the intestinal glands significantly proliferated (day 38). After the third round of DSS administration (day 44), adenomas were observed with dysplastic cells, and later (day 70) tumors developed (**Figure 1F**).

**Figure 2:**
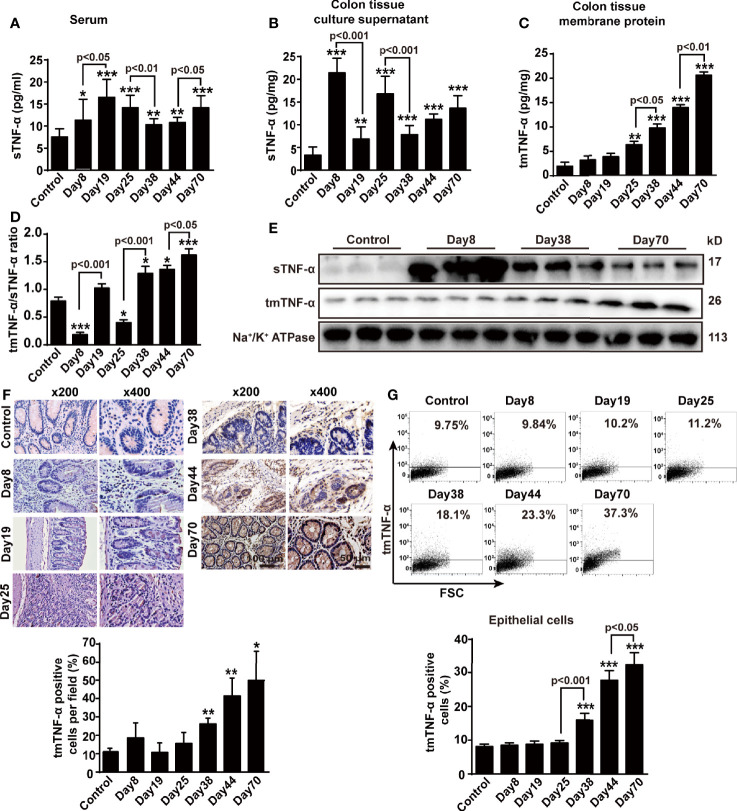
sTNF-α levels are increased at the inflammation stage, but tmTNF-α expression is enhanced at the tumor stage. Mice were treated with AOM and DSS as described in **Figure 1A** (n = 6, each group). **(A)** Serum concentrations of sTNF-α detected by ELISA. Levels of sTNF-α released in supernatants **(B)** and tmTNF-α expression in the membrane protein **(C)** of 24-h-cultured colonic tissues detected by ELISA and their ratios **(D)**. **(E)** Western blot analysis of sTNF-α in colonic homogenates and tmTNF-α in colonic membrane protein. **(F)** Representative immunohistochemical staining for intracolonic expression of tmTNF-α (x200, ×400) and their quantitative data. **(G)** Representative cytogram for tmTNF-α expression on colonic epithelial cells isolated from AOM/DSS-treated mice analyzed by flow cytometry and their quantitative data. All quantitative data are expressed as means ± SEM, *P < 0.05, **P < 0.01, ***P < 0.001 versus control.

In this animal model, serum sTNF-α levels increased gradually and peaked on day 19, followed by a relative decrease, but slightly raised again at the tumor stage (**Figure 2A**). We cultured colon tissue for 24 h to measure the levels of sTNF-α released in the supernatant and tmTNF-α in the membrane proteins using an ELISA in order to accurately evaluate and compare the changes between the levels of both forms of TNF-α in the colon tissue. sTNF-α levels increased significantly in mice with DSS-induced inflammation but then declined during 2-week intervals, and were enhanced again to a certain degree at the tumor stage (**Figure 2B**). In contrast, tmTNF-α levels remained unchanged and began to increase in the second round of inflammation and peaked at the tumor stage (**Figure 2C**). The ratio of tmTNF-α to sTNF-α was approximately 0.85 in the normal group but decreased significantly during the first and second rounds of DSS consumption (**Figure 2D**), indicating that sTNF-α predominated in the inflammation phases. In contrast, this ratio was significantly increased at the uncontrolled inflammation stage and the tumor stage, suggesting that increased tmTNF-α expression was associated with malignant transformation. Western blot analysis showed similar results: intracolonic sTNF-α levels increased dominantly in the inflammation phase, but tmTNF-α levels in the membrane protein fraction were significantly elevated in the tumor phase (**Figure 2E**). Immunohistochemical staining using a tmTNF-α-specific antibody that is not cross reactive to sTNF-α (28) revealed that tmTNF-α was expressed on infiltrated leukocytes on day 38 but expressed on glandular epithelial cells or tumor cells in addition to infiltrated leukocytes in the tumor stage (**Figure 2F**). We further isolated diseased colonic epithelial cells and found that tmTNF-α expression did not significantly increase in colonic epithelial cells until malignant transformation and peaked in the tumor stage (**Figure 2G**).

### The tmTNF-α/sTNF-α Ratio Is Associated With the Accumulation of MDSCs and Treg Cells in a Mouse AOM/DSS-Induced CAC Model But Negatively Correlates With Macrophages

**Figure 3:**
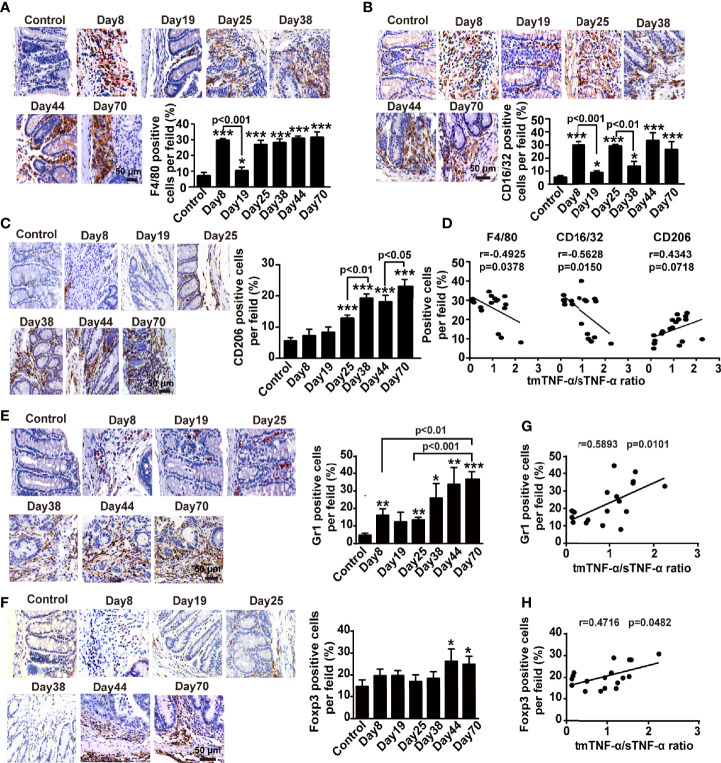
The tmTNF-α/sTNF-α ratio negatively correlates with the accumulation of macrophages but positively associates with the infiltration of MDSCs and Treg cells in the colonic tissue of CAC. Mice were treated with AOM and DSS as described in **Figure 1A** (n = 6-8, each group). Representative immunohistochemistry images of F4/80^+^ macrophages **(A),** CD16/32^+^ M1 type **(B)** or CD206^+^ M2 type macrophages **(C),** Gr1^+^ MDSCs **(E)** and Foxp3^+^ Treg cells **(F)** infiltrated in colonic tissues (×400) and their quantitative data. The correlation of the tmTNF-α/sTNF-α ratio with percentages of F4/80^+^ macrophages, CD16/32^+^ M1 type or CD206^+^ M2 type macrophages **(D)**, MDSCs **(G)** and Treg cells **(H)** (n = 18). All quantitative data are expressed as means ± SEM. *P < 0.05, **P < 0.01, ***P < 0.001 versus control.

Next, we measured changes in immune cells, such as macrophages and Th17 cells, and immunosuppressive cells, including CD4^+^CD25^+^ regulatory T cells (Tregs) and myeloid-derived suppressor cells (MDSCs), using flow cytometry and immunohistochemistry to determine potential relationships between both forms of TNF-α and these cells in the mouse AOM/DSS-induced CAC model. The number of F4/80^+^ macrophages increased during inflammation and peaked on day 19 and day 44, but returned to baseline levels in the spleen and mesenteric lymph nodes at the tumor stage (**Supplementary Figures S1A, B**). However, in the colonic tissue, infiltrated F4/80^+^ macrophages were detected at approximately all time-points, except the first interval in which the infiltration significantly decreased (**Figure 3A**). Moreover, the number of CD16/32^+^ M1 macrophages significantly increased when mice drank DSS-containing water but decreased during the intervals (**Figure 3B**), while the number of CD206^+^ M2 type macrophages began to increase on day 25 and peaked at the tumor stage (**Figure 3C**). The number of CD4^+^CD17A^+^Th17 cells with proinflammatory and tumor-promoting effects increased slightly and peaked on day 19 in the spleen, but did not increase until day 70 in the mesenteric lymph nodes (**Supplementary Figures S1C, D**), while the number of Th17 cells in the colonic tissue was significantly enhanced on day 25 and peaked on day 70 (**Supplementary Figure S1E**). Importantly, the ratio of intracolonic tmTNF-α/sTNF-α negatively correlated with the infiltrated F4/80^+^ macrophages and M1 macrophages in the colon (**Figure 3D**), but not with M2 macrophages or Th17 cells (**Figure 3D** and **Supplementary Figure S1F**).

The number of CD11b^+^Gr1^+^MDSCs, a heterogeneous population of immature myeloid cells with a remarkable ability to suppress T cell responses that are characterized by co-expression of CD11b and GR1 in mice (32–34), peaked on day 19 in the spleen and on day 25 in mesenteric lymph nodes and then decreased significantly with disease progression (**Supplementary Figures S1G, H**). In contrast, the number of MDSCs in the colonic tissue was augmented on days 8 and 25 in animals with DSS-induced inflammation, further increased with the disease development, and reached a peak at the tumor stage (**Figure 3E**). The number of another type of immunosuppressive cell, CD4^+^CD25^+^Foxp3^+^Treg cells, increased in the spleen at all time points, except at the tumor stage (**Supplementary Figure S1I**), but the number of Treg cells increased during DSS drinking and returned to baseline levels in mesenteric lymph nodes during intervals between DSS consumption (**Supplementary Figure S1J**). However, a certain number of Treg cells was present in the lamina propria of colonic tissue of normal mice but did not increase during the inflammation stage until malignant transformation (**Figure 3F**). Interestingly, the ratio of intracolonic tmTNF-α/sTNF-α positively correlated with the infiltration of both MDSCs and Treg cells in the colonic tissues (**Figures 3G, H**). Based on these data, tmTNF-α expression in the colon was closely associated with intracolonic infiltration of MDSCs and Treg cells.

### The tmTNF-α/sTNF-α Ratio Is Negatively Correlated With Proinflammatory Mediators but Positively Correlated With the Anti-inflammatory Cytokine IL-10

**Figure 4:**
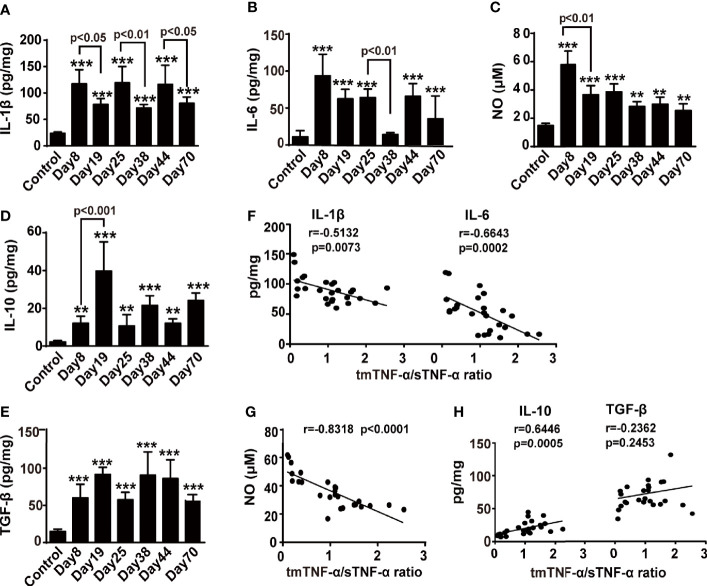
The tmTNF-α/sTNF-α ratio negatively correlates with proinflammatory mediators but positively correlates with IL-10. Mice were treated with AOM and DSS as described in **Figure 1A** (n = 5-8, each group). Concentrations of IL-1β **(A)**, IL-6 **(B)**, IL-10 **(D)**, and TGF-β **(E)** in colonic tissue homogenates detected at indicated time points by ELISA. The levels of NO **(C)** in colonic tissue homogenates measured by Griess method. The correlation of tmTNF-α/sTNF-α ratio with concentrations of IL-1β and IL6 **(F)**, or NO **(G)**, or IL-10 and TGF-β **(H)** [n = 26, except IL-10 (n = 25)]. All values represent the mean ± SEM. **P < 0.01, ***P < 0.001 versus control.

We detected cytokines and NO in colonic tissue homogenates from the AOM/DSS mouse model to analyze the correlations between both forms of tmTNF-α and pro- and anti-inflammatory factors. The concentrations of IL-1β and IL-6 were increased while animals drank DSS-containing water and were reduced in the intervals between DSS consumption (**Figures 4A, B**); the release of NO peaked on day 8 (**Figure 4C**). In contrast, IL-10 and TGF-β levels increased when animals were drinking DSS-containing water compared to the control, but were further enhanced in the intervals between DSS consumption, with the exception of TGF-β at the tumor stage (**Figures 4D, E**). Interestingly, the ratio of tmTNF-α/sTNF-α was negatively correlated with the levels of proinflammatory mediators, including IL-1β, IL-6, and NO (**Figures 4F, G**), but was positively correlated with the level of the anti-inflammatory cytokine IL-10 (**Figure 4H**) in colonic tissues from mice with AOM/DSS-induced CAC. However, this ratio was not associated with the secretion of TGF-β (**Figure 4H**). Our data suggested an association of tmTNF-α with inflammation resolution during disease progression.

### Increasing tmTNF-α Expression by Treatment With an Antibody in the Inflammation Stage Suppresses Inflammation and Tumor Formation

**Figure 5:**
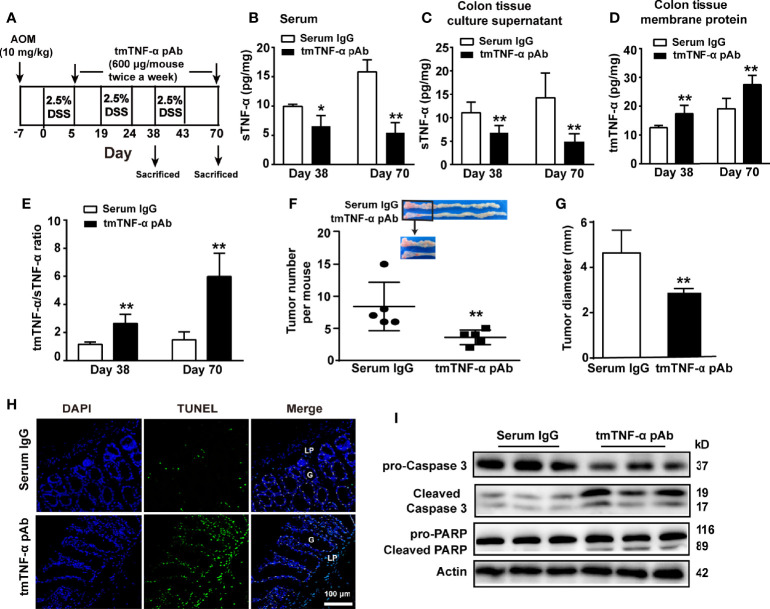
tmTNF-α antibody treatment in the first inflammation stage suppresses tmTNF-α shedding and tumor growth. **(A)** Schematic treatment of mice with tmTNF-α polyclonal antibody (pAb) in AOM/DSS-induced CAC. Mice were intraperitoneally injected with 600 μg of tmTNF-α pAb twice a week, and the treatment was from day 5 to day 70. Normal rabbit serum IgG served as a control. (n = 5, each group) **(B)** Serum concentrations of sTNF-α detected by ELISA. Levels of sTNF-α released in supernatants **(C)** and tmTNF-α expression in the membrane protein **(D)** of 24-h-cultured colonic tissues detected by ELISA and their ratios **(E)** on day 38 and day 70. **(F, G)** Tumor number and size. **(H, I)** Representative images of apoptosis in diseased colons detected by TUNEL (×200), and western blot analysis for cleavage of caspase 3 and PARP on day 70. G: glands; LP: lamina propria. All quantitative data represent the mean ± SEM. *P < 0.05, **P < 0.01 versus serum IgG.

Our results indicate an association of tmTNF-α with the inhibition of inflammation; however, tmTNF-α expression began to increase in the later stage of colitis. If the tmTNF-α expression time window can be shifted beforehand to the early stage of inflammation, disease progression may be interrupted. Previously, we developed a monoclonal antibody specific to human tmTNF-α and a polyclonal antibody specific to murine tmTNF-α. Both antibodies inhibit tmTNF-α shedding by competing with TACE that is responsible for tmTNF-α processing (28), increasing tmTNF-α expression and decreasing sTNF-α release. To test our hypothesis, mice were intraperitoneally injected with 600 μg of the murine tmTNF-α polyclonal antibody twice a week beginning on the day after the first cycle of drinking DSS water (**Figure 5A**). The effects of the antibody were detected at the inflammation stage on day 38 and at the tumor stage on day 70. Indeed, the antibody decreased sTNF-α levels in serum and cultured colonic tissue supernatant (**Figures 5B, C**), while the antibody increased tmTNF-α expression in colonic tissues (**Figure 5D**) with an elevated ratio of intracolonic tmTNF-α/sTNF-α (**Figure 5E**) observed on day 38 and on day 70. In addition, immunohistochemical staining revealed that the antibody increased tmTNF-α expression in both infiltrated cells and glandular epithelial cells on day 38 and day 70 (**Supplementary Figure S2A**). Importantly, antibody treatment remarkably suppressed the formation of inflammation-associated tumors, as the number and size of tumors were significantly reduced (**Figures 5F, G**). TUNEL staining showed that the antibody induced apoptosis mainly in tumor cells (**Figure 5H**), and the western blot analysis of diseased colonic tissue revealed that the antibody induced caspase 3 activation and cleavage of its substrate PARP (**Figure 5I**).

**Figure 6:**
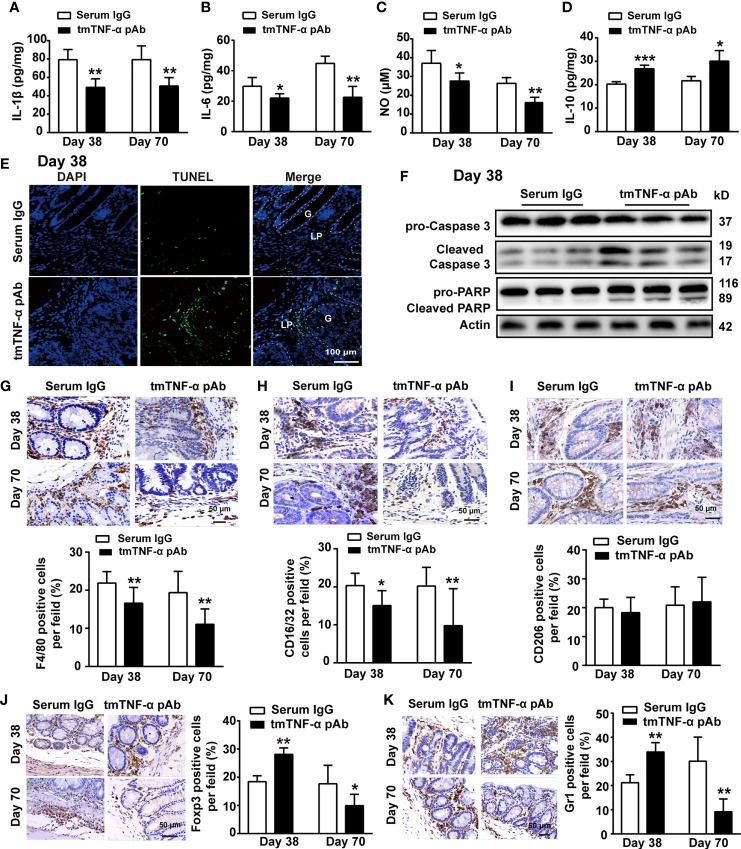
tmTNF-α antibody treatment in the first inflammation stage inhibits inflammation. tmTNF-α pAb was administered in the inflammation stage as described in **Figure 5A** (n = 5, each group). Concentrations of IL-1β **(A)**, IL-6 **(B),** and IL-10 **(D)** in colonic tissue homogenates detected by ELISA. The levels of NO **(C)** in colonic tissue homogenates measured by Griess method. **(E, F)** Representative images of apoptosis in diseased colons detected by TUNEL (×200), and the western blot analysis for cleavage of caspase 3 and PARP on day 38. G: glands; LP: lamina propria. **(G–K)** Representative immunohistochemistry images of F4/80^+^ Macrophages, CD16/32^+^ type 1 or CD206^+^ type 2 macrophages, Foxp3^+^ Tregs and Gr1^+^ MDSCs in colonic tissues (×400) and their quantitative data. All quantitative data represent the mean ± SEM. *P < 0.05, **P < 0.01, ***P < 0.001 versus serum IgG.

Next, we observed whether the antibody suppressed inflammation in the inflammation stage on day 38 and the tumor stage on day 70. Although a significant improvement in weight loss or DAI score was not observed (**Supplementary Figures S2B, C**), treatment with the tmTNF-α antibody significantly inhibited the production of proinflammatory mediators, including IL-1β, IL-6, and NO, but prompted the release of the anti-inflammatory cytokine IL-10 in colonic tissue homogenates at both the inflammation and tumor stages (**Figures 6A–D**). Interestingly, the antibody induced apoptosis mainly in infiltrated cells on day 38 (**Figures 6E, F**), which might contribute to inhibiting inflammation. Furthermore, the tmTNF-α antibody markedly reduced the intracolonic infiltration of F4/80^+^ macrophages and M1 type macrophages (**Figures 6G, H**), rather than M2 type macrophages (**Figure 6I**) at inflammation and tumor stages. However, the antibody promoted the infiltration of immunosuppressive Treg cells and MDSCs at the inflammation stage but reduced their accumulation at the tumor stage (**Figures 6J, K**). The data indicate that the tmTNF-α antibody not only suppressed inflammation at the inflammation stage but also inhibited tumor-associated inflammation.

In addition, we treated mice with tmTNF-α antibody twice a week starting at the end of the second cycle of drinking DSS water (**Supplementary Figure S3A**), and found that the tmTNF-α antibody inhibited tmTNF-α shedding, decreased sTNF-α levels in the serum and cultured colonic tissue supernatant (**Supplementary Figures S3B, C**), increased the expression of the transmembrane molecule in the diseased colonic tissue (**Supplementary Figures S3D, F**), and increased the ratio of intracolonic tmTNF-α/sTNF-α (**Supplementary Figure S3E**). The tmTNF-α antibody also remarkably reduced the number and size of tumors and increased apoptosis in both tumor cells and infiltrated leukocytes (**Supplementary Figures S3G–I**) through the activation of caspase 3 and cleavage of PARP (**Supplementary Figure S3J**). Similarly, the tmTNF-α antibody suppressed the production of proinflammatory mediators (**Supplementary Figures S4A–C**), promoted the release of IL-10 (**Supplementary Figure S4D**), and inhibited the infiltration of macrophages, Treg cells, and MDSCs at the tumor stage (**Supplementary Figures S4E–G**). Thus, suppressing tmTNF-α processing at the inflammation stage might attenuate inflammation and limit subsequent tumor formation.

### Treatment With the tmTNF-α Antibody at the Tumor Stage Suppresses Tumor Growth

**Figure 7:**
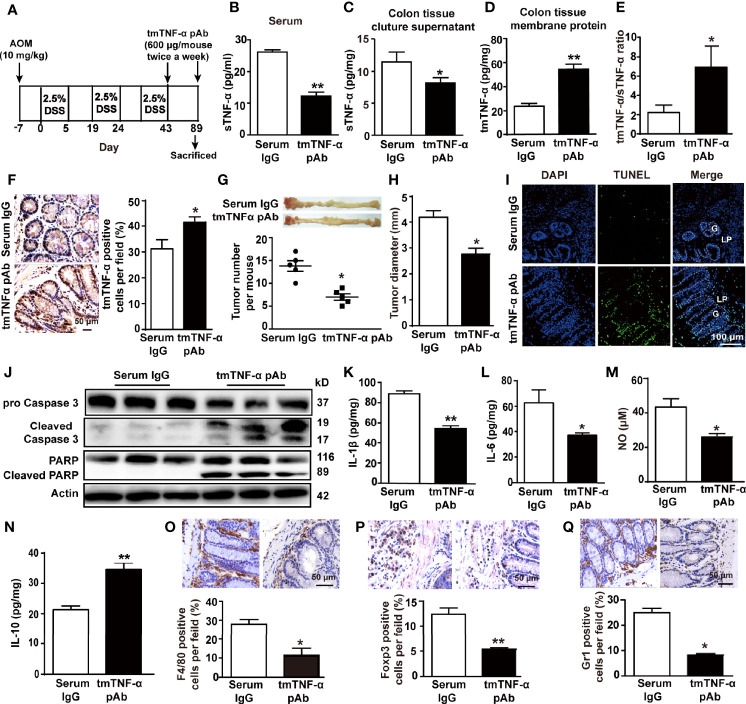
tmTNF-α antibody treatment in the tumor stage inhibits inflammation and tumor growth. **(A)** Schematic treatment of mice with tmTNF-α pAb in AOM/DSS-induced CAC. Mice were intraperitoneally injected with 600 μg of tmTNF-α pAb twice a week, and the treatment was from day 43 to day 89. Normal rabbit serum IgG served as a control (n = 5, each group). **(B)** Serum concentrations of sTNF-α detected by ELISA. Levels of sTNF-α released in supernatants **(C)** and tmTNF-α expression in the membrane protein **(D)** of 24-h-cultured colonic tissues detected by ELISA and their ratios **(E).** Representative immunohistochemical staining of tmTNF-α positive cells in colons (×400) and their quantitative data **(F)**. **(G, H)** tumor number and size. **(I, J)** Representative images of apoptosis in colonic tissues detected by TUNEL (×200), and western blot analysis for cleavage of caspase 3 and PARP. G: glands; LP: lamina propria. Concentrations of IL-1β **(K)**, IL-6 **(L)** and IL-10 **(N)** in colonic tissue homogenates detected by ELISA. The levels of NO **(M)** in colonic tissue homogenates measured by Griess method. **(O–Q)** Representative immunohistochemistry images of F4/80^+^ Macrophages, Foxp3^+^ Tregs and Gr1^+^ MDSCs in colonic tissues (×400) and their quantitative data. All quantitative data represent the mean ± SEM. *P < 0.05, **P < 0.01 versus serum IgG.

To observe the effect of the antibody on CRC, we treated mice with the tmTNF-α antibody twice a week starting at the end of the third cycle of drinking DSS-containing water (**Figure 7A**). Administration of the tmTNF-α antibody decreased sTNF-α release (**Figures 7B, C**) and increased tmTNF-α expression (**Figures 7D, F**) with a raised ratio of tmTNF-α/sTNF-α in colonic tissue (**Figure 7E**). The antibody remarkably reduced the number and size of tumors (**Figures 7G, H**) by promoting apoptosis (**Figures 7I, J**). Moreover, the tmTNF-α antibody significantly inhibited the production of IL-1β, IL-6, and NO and increased IL-10 release in colonic tissue homogenates (**Figures 7K–N**). The antibody also significantly reduced the intracolonic infiltration of macrophages, Tregs, and MDSCs (**Figures 7O–Q**). The data indicate an antitumor effect of the antibody through the induction of apoptosis and inhibition of inflammation.

### tmTNF-α, Rather Than sTNF-α, Actively Suppresses LPS-Induced Production of Inflammatory Mediators

**Figure 8:**
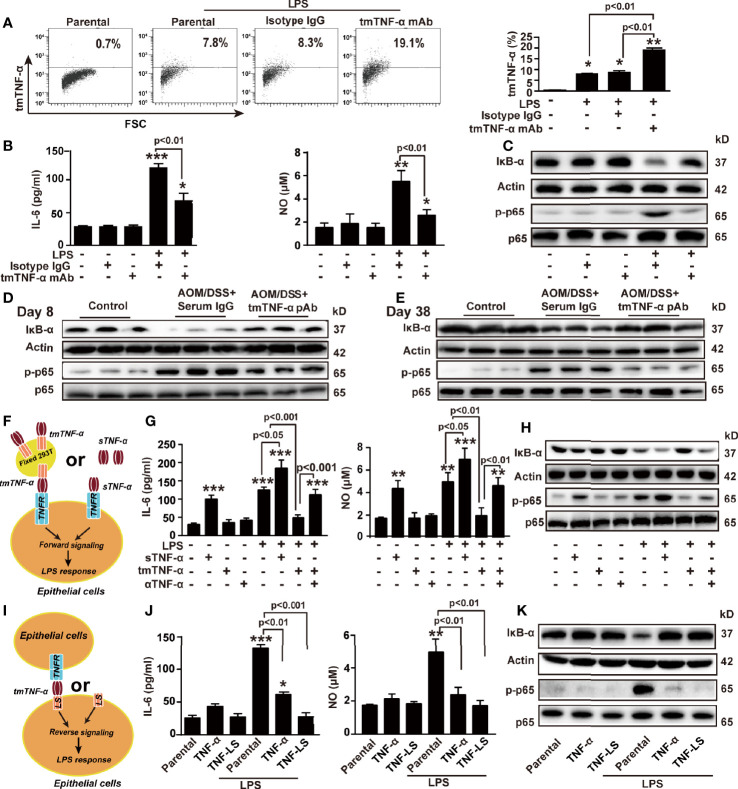
tmTNF-α actively suppresses LPS-induced production of inflammatory mediators *via* dual signaling. NCM460 cells were stimulated with 10 ng/ml LPS combined with 2 μg/ml of tmTNF-α mAb for 6 h. Isotype IgG served as a control. **(A)** tmTNF-α expression on the cell surface assessed by flow cytometry and quantitative data. **(B)** Concentrations of IL-6 and NO in culture supernatants at 10 h after stimulation. **(C)** Representative western blot of three independent experiments for IκBα degradation and p65 phosphorylation at 1 h after stimulation. **(D, E)** Mice were intraperitoneally injected with 600 μg tmTNF-α pAb twice a week starting on day 5 followed by AOM/DSS treatment. Normal rabbit serum IgG served as a control. Western blot analysis of the NF-κB pathway on day 8 and day 38. **(F–H)** NCM460 cells were cocultured with 100 ng/ml sTNF-α or tmTNF-α stably expressed on the cell surface of fixed 293T cells at a ratio of 1:10 in the presence of 10 ng/ml LPS **(F)**. Concentrations of IL-6 and NO in culture supernatants at 10 h after stimulation **(G)**, and representative western blot of three independent experiments for the NF-κB pathway at 1 h after stimulation **(H)**. **(I–K)** TNF-α and TNF-LS stably transfected NCM460 cells **(I)** and their parental cells were stimulated with 10 ng/ml LPS for 10 h **(J)** Levels of IL-6 and NO. **(K)** Representative western blot of three independent experiments for the NF-κB pathway at 1 h after LPS stimulation. All quantitative data represent as means ± SEM of four independent experiments. *P < 0.05, **P < 0.01, ***P < 0.001 versus control for **(A, B, G)**, versus parental for **(J)**.

To further explore the mechanism by which the tmTNF-α antibody exerted anti-inflammatory effects and therefore inhibited inflammation-associated cancer, a human colon mucosal epithelial cell line NCM460 was stimulated with LPS (10 ng/ml) to induce tmTNF-α expression. LPS-induced tmTNF-α expression at 6 h was significantly enhanced by treatment with the human tmTNF-α monoclonal antibody (mAb, **Figure 8A**). Consistent with the results obtained from the mouse model, the antibody effectively suppressed LPS-induced mRNA expression of proinflammatory factors, including *IL-6* and *iNOS* (**Supplementary Figure S5A**), and the release of IL-6 and NO (**Figure 8B**). In addition, LPS-induced IκBα degradation and phosphorylation of NF-κB p65 were effectively blocked by the antibody (**Figure 8C**). In line with the *in vitro* results, treatment of AOM/DSS mice with the tmTNF-α antibody significantly inhibited IκBα degradation and phosphorylation of NF-κB p65 on day 8 and day 38 (**Figures 8D, E**), indicating that the anti-inflammatory effects of the tmTNF-α antibody were mediated by suppressing the NF-κB pathway.

The suppressive effect of the tmTNF-α antibody on the LPS response might be due to increased tmTNF-α expression and reduced sTNF-α release. We stably transfected 293T cells with tmTNF-α (**Supplementary Figure S5B**) and fixed these cells as effector cells to determine whether tmTNF-α was responsible for this anti-inflammatory effect of the antibody. tmTNF-α-overexpressing cells or sTNF-α were added to NCM460 cells to compare the effect of the two forms of exogenous TNF-α on the LPS response (**Figure 8F**). As expected, LPS-induced expression of *IL-6* mRNA and *iNOS* mRNA (**Supplementary Figure S5C**) and release of IL-6 and NO (**Figure 8G**) were suppressed by tmTNF-α but increased by sTNF-α. Additionally, LPS-induced activation of NF-κB was also suppressed by tmTNF-α but promoted by sTNF-α (**Figure 8H**).

Since tmTNF-α functions as a receptor and transmits outside-to-inside (reverse) signals to tmTNF-α-bearing cells, we transferred the full-length gene encoding TNF-α or its leading sequence (LS) mutant into NCM460 cells. The transfectants overexpressed tmTNF-α and TNF-LS on the cell surface (**Supplementary Figure S5D**). TNF-α-overexpressing cells secreted high levels of sTNF-α; however, TNF-LS-overexpressing cells did not secrete sTNF-α (**Supplementary Figure S5E**) because of the lack of an extracellular sTNF-α segment. Therefore, TNF-LS cannot bind to the TNF-α receptor (excluding forward signaling) but retains the intracellular segment of tmTNF-α for reverse signaling (**Figure 8I**). Interestingly, LPS-induced IL-6 and NO production and activation of NF-κB were significantly inhibited by the transfection of TNF-α but completely blocked by the transfection of the TNF-LS mutant (**Supplementary Figure S5F** and **Figures 8J, K**). Although TNF-α transfectants secreted a large amount of sTNF-α, tmTNF-α overexpression still blocked the LPS response of colonic epithelial cells. The inhibitory effect of TNF-LS indicates an active anti-inflammatory role of tmTNF-α-mediated reverse signaling. Similar phenomena were observed in another human colon mucosal epithelial cell line, HCoEpiC (**Supplementary Figures S6A-H**).

## Discussion

**Figure 9:**
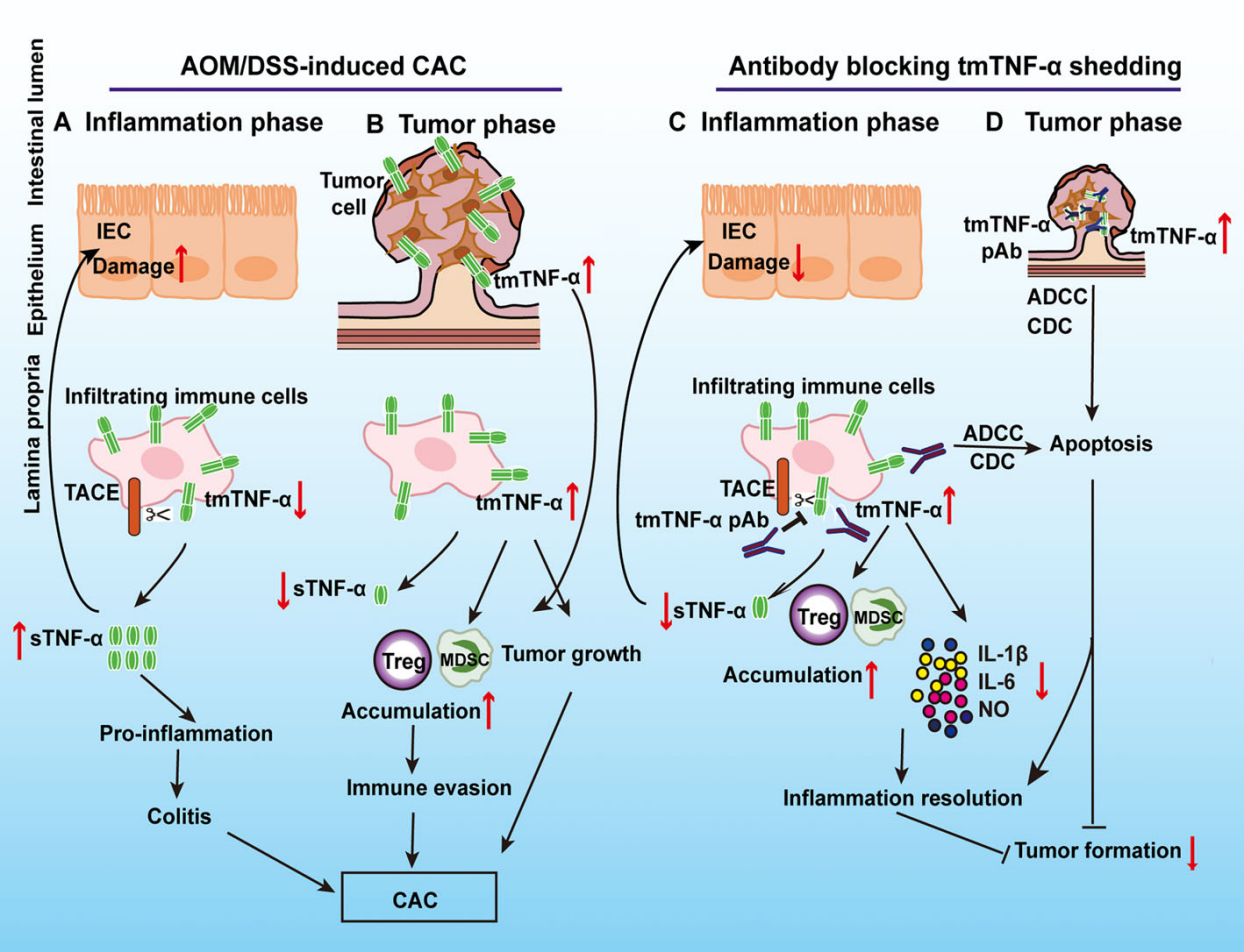
Schematic summary of the protective effect of tmTNF-α on CAC by a specific antibody that prevents tmTNF-α shedding. In AOM/DSS-induced CAC, tmTNF-α is rapidly processed into sTNF-α that is mainly released by inflammatory cells in the inflammation phase to promote colitis **(A)**, while tmTNF-α expression is increased on epithelial cells in addition to infiltrated leukocytes in malignant transformation to facilitate CAC development. Increased tmTNF-α expression promotes the accumulation of MDSCs and Treg cells, which leads to immune evasion **(B)**. Using a specific antibody to prevent tmTNF-α shedding and increase tmTNF-α expression in the inflammation phase inhibits the production of inflammatory mediators (IL-1β, IL-6, and NO) and the infiltration of M1 macrophages, induces apoptosis of inflammatory cells, and promotes intracolonic accumulation of MDSCs and Treg cells **(C)**. As a result, inflammation resolution is facilitated and subsequent tumor formation is suppressed. In addition, the antibody induces apoptosis of tmTNF-α-positive tumor cells by ADCC and CDC **(D)**.

Here, we described dynamic changes in the ectodomain shedding of tmTNF-α during AOM/DSS-induced CAC, namely, the processing of tmTNF-α was markedly increased during the inflammation phase but decreased during malignant transformation (**Figures 9A, B**). The ratio of intracolonic tmTNF-α/sTNF-α was associated with increased anti-inflammatory responses and decreased release of pro-inflammatory mediators, indicating an anti-inflammatory effect of tmTNF-α. The administration of a tmTNF-α antibody that prevents the ectodomain shedding of tmTNF-α at the inflammation stage significantly suppressed inflammation and subsequent tumor formation (**Figures 9C, D**).

Although TNF-α expression is increased at the mRNA and protein levels in IBD and AOM/DSS-induced CAC (12), the changes in TNF-α at the posttranslational level in this pathological process remain unclear. Our results originally revealed that sTNF-α levels were increased in the inflammation phase, while tmTNF-α levels were enhanced during malignant transformation and peaked in the tumor phase. These findings suggest that tmTNF-α was rapidly processed into sTNF-α that promoted colitis, since sTNF-α, a pro-inflammatory cytokine, promotes colonic inflammation by inducing the expression of IL-1β and IL-6 in colonic epithelial cells through activating the NF-κB pathway (35). In contrast, the ectodomain shedding of tmTNF-α was decreased during malignant transformation, as evidenced by the elevated tmTNF-α expression levels and reduced sTNF-α release in the colonic tissue. Consequently, the ratio of tmTNF-α/sTNF-α was significantly increased. Interestingly, the ratio of tmTNF-α/sTNF-α was positively correlated with the production of the anti-inflammatory cytokine IL-10 and intracolonic infiltration of immunosuppressive MDSCs and Treg cells but negatively correlated with the release of inflammatory mediators, including IL-1β, IL-6, and NO, and M1 macrophage accumulation, suggesting an anti-inflammatory effect of tmTNF-α. As shown in our previous study and studies from other researchers, tmTNF-α functions in the resolution of inflammation by suppressing LPS/TLR4 signaling and the production of IL-1β and IL-6 in macrophages (28, 36). Furthermore, in addition to the different expression windows of both forms of TNF-α during CAC development, the cell types expressing these proteins were not quite the same. sTNF-α has been reported to be mainly released from infiltrated myeloid cells (12), and our results revealed that tmTNF-α was expressed at high levels on enterocytes, including intestinal adenomatous cells, in addition to infiltrated immune cells. Notably, tmTNF-α expression by colonic epithelial cells did not increase until the precancerous stage, indicating that it might be a biomarker for the malignant transformation of CAC. Based on our findings and those from other researchers, tmTNF-α expressed by tumor cells not only promotes proliferation, transformation, chemoresistance, and metastasis (31, 37, 38) but also facilitates immune evasion by promoting the suppressive activities of MDSCs and accumulation of Treg cells (39, 40). tmTNF-α expression in intestinal epithelial cells likely facilitates malignant transformation and tumor formation. Therefore, tmTNF-α functions as a double-edged sword, an anti-inflammatory molecule and a tumor promoter, in CAC.

Although tmTNF-α exerts an anti-inflammatory effect, its expression was too low because of high-level processing of tmTNF-α in the inflammation phase. We assumed that if the time window of tmTNF-α expression was shifted ahead to the inflammation stage, malignant transformation might be suppressed. We used a specific antibody that prevents tmTNF-α ectodomain shedding to treat mice in the inflammation phase and successfully increased tmTNF-α expression and decreased sTNF-α release, along with a raised tmTNF-α/sTNF-α ratio. As expected, the antibody significantly reduced the infiltration of M1 macrophages and the production of IL-1β, IL-6, and NO but promoted IL-10 release and the accumulation of Treg cells and MDSCs in the diseased colonic tissue during the inflammation phase. Importantly, the subsequent tumor formation was effectively inhibited, as the number and size of the tumors were significantly decreased. The benefit of the antibody was attributed to increased tmTNF-α expression that suppressed the release of inflammatory mediators. This was supported by the following evidence: 1) The antibody increased tmTNF-α expression and exerted anti-inflammatory effects by inhibition of the NF-κB pathway *in vivo* and *in vitro*; 2) Direct addition of exogenous tmTNF-α or sTNF-α to the culture of colonic epithelial cell lines NCM460 and HCoEpiC led to the opposite effects on the response to LPS *via* TNFR. In contrast to the proinflammatory effect of sTNF-α, tmTNF-α actively suppressed LPS-induced activation of the NF-κB pathway and production of IL-6 and NO, indicating tmTNF-α-induced LPS resistance through its forward signaling; and 3) Transfection of TNF-α or TNF-LS (lack of sTNF-α and TNFR binding but retention of the intracellular fragment of tmTNF-α to transduce reverse signaling) into NCM460 and HCoEpiC suppressed LPS-induced activation of the NF−κB pathway and production of IL-6 and NO, indicating tmTNF-α-induced LPS resistance through its reverse signaling. Second, the anti-inflammatory effect of the tmTNF-α antibody was activation of immunosuppressive cells by increased tmTNF-α expression. This was supported by our original evidence that the antibody significantly promoted intracolonic infiltration of MDSCs and Tregs at the inflammation stage. We have previously found that MDSCs exert a cardioprotective effect in heart failure (41) and tmTNF-α promotes suppressive activities of MDSCs (39). Furthermore, tmTNF-α is a primary ligand for TNFR2 (42) and the interaction of tmTNF-α with TNFR2 results in activation of Tregs and induction of their expansion (40). Thus, the resolution of inflammation through tmTNF-α was the main mechanism by which the antibody suppressed tumor formation in the mouse AOM/DSS-induced CAC model.

Another important mechanism of the tmTNF-α antibody was the induction of apoptosis. Our results revealed that the antibody induced apoptosis not only in infiltrated cells but also in tumor cells; the former led to inflammation resolution and the latter resulted in inhibition of tumor formation and growth. The tmTNF-α density on leukocytes is associated with the primary response to infliximab, including apoptosis of peripheral blood mononuclear cells in IBD. Additionally, infliximab induces apoptosis of monocytes from patients with active Crohn’s disease in a caspase-dependent manner (20). Namely, anti-TNF-α agents, such as infliximab, can bind to tmTNF-α and induce apoptosis of tmTNF-α-bearing cells *via* reverse signaling (43). Although our antibody is unable to directly activate tmTNF-α-mediated reverse signaling as its recognized epitope does not exist in TNFR binding site, it was possible that sTNFR or membrane TNFR, as a ligand, bound to tmTNF-α (as a receptor) that was upregulated by the antibody, which activated tmTNF-α-mediated reverse signaling, and thus induced apoptosis. Moreover, our previous study documented that the tmTNF-α antibody is cytotoxic to tmTNF-α-expressing breast cancer cells *via* antibody-dependent cell-mediated cytotoxicity (ADCC) and complement-dependent cytotoxicity (CDC) (31). In this study, antibody treatment during the tumor stage also effectively suppressed the growth of tmTNF-α-expressing adenoma by inducing apoptosis and inhibiting tumor-associated inflammation.

In summary, in a mouse AOM/DSS-induced CAC model, sTNF-α was mainly released in the inflammation phase to promote colitis, while tmTNF-α was expressed by adenoma cells to facilitate tumor development. We have not yet clearly determined whether this phenomenon also exists in patients with CAC, although a report has shown that tmTNF-α is expressed in colorectal cancer (37). Since tmTNF-α functions as a double-edged sword in AOM/DSS-induced CAC, changing its expression window to the inflammation stage may benefit the patients by promoting inflammation resolution *via* tmTNF-α-mediated signaling. The tmTNF-α antibody that targets tmTNF-α processing, unlike selective inhibitors of sTNF-α, anti-TNF antibodies, or soluble TNFR, not only reduced sTNF-α levels but also increased tmTNF-α expression and thus effectively prevented AOM/DSS-induced CAC through the inhibition of inflammation and induction of apoptosis, which provides a new insight into the blockade of this pathological process.

## Data Availability Statement

The original contributions presented in the study are included in the article/**Supplementary Material**. Further inquiries can be directed to the corresponding authors.

## Ethics Statement

The animal study was reviewed and approved by the Ethics Committee of Tongji Medical College of HUST.

## Author Contributions

HB, BL, RJ, MZ, and XZ planned and performed the experiments. HB and BL analyzed the data. ZL, BY, and JW initiated the project and designed the study. XZ and ZL drafted and wrote the manuscript. All authors contributed to the article and approved the submitted version.

## Funding

This study was supported by the National Natural Science Foundation of China (Major research program 91029709 and General program 31671470 to ZL) and by China Postdoctoral Science Foundation (2018M642852 to HB).

## Conflict of Interest

The authors declare that the research was conducted in the absence of any commercial or financial relationships that could be construed as a potential conflict of interest.

## Publisher’s Note

All claims expressed in this article are solely those of the authors and do not necessarily represent those of their affiliated organizations, or those of the publisher, the editors and the reviewers. Any product that may be evaluated in this article, or claim that may be made by its manufacturer, is not guaranteed or endorsed by the publisher.
