# Supplementary material for: Suppression of Transmembrane Tumor Necrosis Factor Alpha Processing by a Specific Antibody Protects Against Colitis-Associated Cancer
Source: Front Immunol. 2021 Oct 5;12:687874. doi: 10.3389/fimmu.2021.687874 (PMC8524043; doi:10.3389/fimmu.2021.687874)
Supplement: Supplementary file 1 [file DataSheet_1.pdf]

Suppression of transmembrane tumor necrosis factor alpha processing by a specific antibody protects against colitis-associated cancer

Hongping Ba[1], Rui Jiang[1], Meng Zhang[1], Bingjiao Yin[1], Jing Wang[1], Zhuoya Li[1], Baihua Li[1§] and Xiaoxi Zhou[2§]

[1]Department of Immunology, Tongji Medical College, Huazhong University of Science and Technology, Wuhan, China

[2]Department of Hematology, Tongji Hospital, Huazhong University of Science and Technology, Wuhan, China

Supplementary Table 1. Sequences of primers

Gene	Primers	Length
IL-6[1]	F:5' -GGA TTCAA TGAGGAGACTTGC-3'	197 bp
	R:5' -GTTGGGTCAGGGGTGGTTAT-3'	
iNOS[1]	F:5' -AAGCCCAAGGTCTATGTTCAGG-3’	254 bp
	R:5' -TCGTAAGGAAATACAGCACCAAAG-3’	
GAPDH[1]	F:5’-CAGTCCA TGCCATCACTGCCACCCAG-3’	303 bp
	R: 5’-CAGTGTAGCCCAGGATGCCCTTGAG-3’	
TNF-α[2]	F:5’-cgggatccATGAGCACTGAAAGCATGATCCGGGA-3’	702 bp
	R:5’-ccgctcgagTCACAGGGCAATGATCCCAAAGTAGAC-3’	
TNF-LS[2]	F:5’ –cgggatccATGAGCACTGAAAGCATGATCCGGGA-3’	231 bp
	R:5’-ccgctcgagCTATGCCTGGGCCAGAGGGCTGATTAGA-3	

1. The primers of *IL-6*, *iNOS* and *GAPDH* were used for Real-time PCR
2. The primers of *TNF-α* and *TNF-LS* were used for clone.

F: Forward; R: Reverse

Supplementary Figures

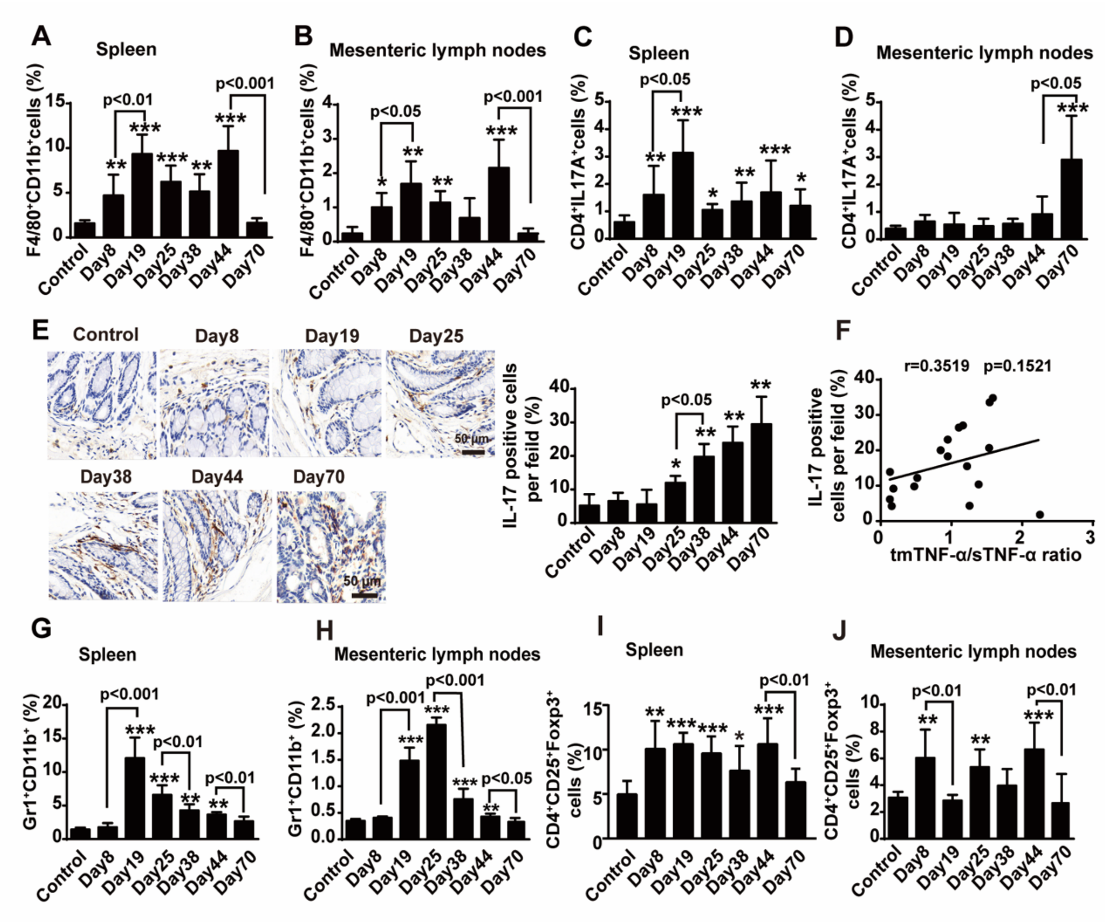

Figure S1. The changes of immune cells in AOM/DSS induced CAC. Mice were intravenously administered with AOM (10 mg/Kg) at day -7, followed by three cycles of drinking 2.5% DSS-containing water for 5 days with interval of 14 days (n = 6-8, each group). Percentages of F4/80$^+$CD11b$^+$ macrophages **(A, B)**, CD4$^+$CD17$^+$Th17 **(C, D)** in the spleen and mesenteric lymph nodes detected at indicated time points by flow cytometry. **(E)** Representative immunohistochemistry images of IL-17$^+$ Th17 cells infiltrated in colonic tissues (×400) and their quantitative data. **(F)** The correlation of the tmTNF-α/sTNF-α ratio with percentages of IL-17$^+$ Th17 in colonic tissues (n = 18). Percentages of Gr1$^+$CD11b$^+$ MDSCs **(G, H)** and CD4$^+$CD25$^+$Foxp3$^+$ Treg cells **(I, J)** in

the spleen and mesenteric lymph nodes detected at indicated time points by flow cytometry. All quantitative data are expressed as means ± SEM. *P < 0.05, **P < 0.01, ***P < 0.001 versus control.

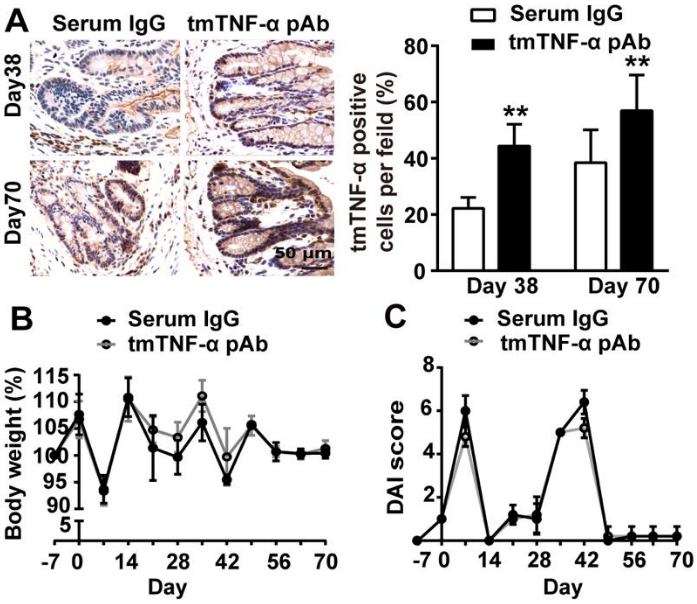

Figure S2. tmTNF-α antibody treatment has no effect on body weight and DAI score**.** Mice were intraperitoneally injected with 600 μg of tmTNF-α pAb twice a week, and the treatment was from day 5 to day 70. Normal rabbit serum IgG served as a control. (n = 5, each group). **(A)** Representative immunohistochemistry images of tmTNF-α expression in colonic tissues (×400) and quantitative data. Time course for body weight **(B)** and disease activity index (DAI) **(C)**. All quantitative data are expressed as means ± SEM, **P < 0.01 versus serum IgG.

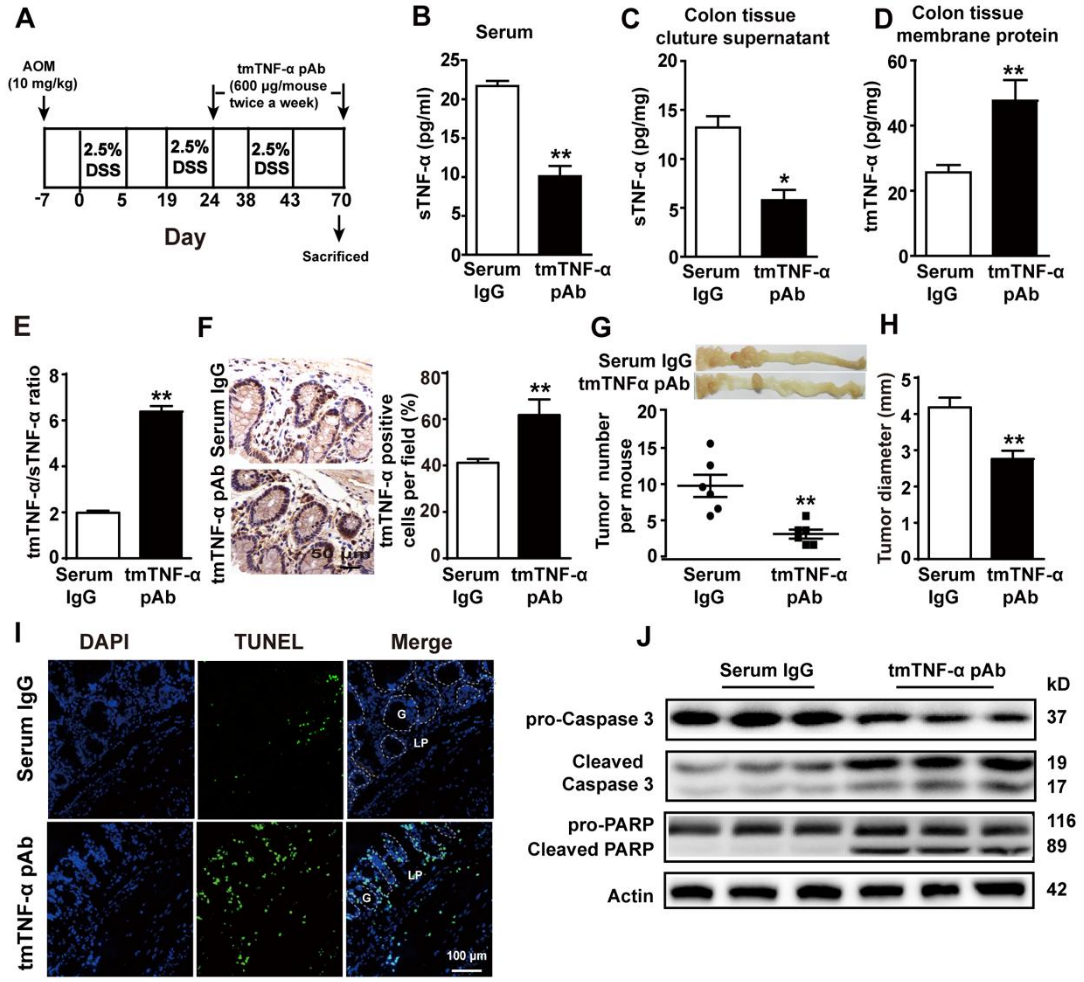

Figure S3. tmTNF-α Ab treatment in the repeated inflammation stage inhibits tumor formation. **(A)** Schematic treatment of mice with tmTNF-α pAb in AOM/DSS-induced CAC. Mice were intraperitoneally injected with 600 μg of tmTNF-α pAb or normal serum IgG twice a week, and the treatment was from day 24 to day 70 (n = 5, each group). **(B)** Concentrations of serum sTNF-α detected by ELISA. Concentrations of sTNF-α in supernatants **(C)** and tmTNF-α expression in the membrane protein **(D)** of 24-h cultured colonic tissues detected by ELISA and their ratios **(E)**. **(F)** Representative immunohistochemical staining of tmTNF-α positive cells in colons (×400) and their quantitative data. **(G, H)** Tumor number and size. **(I, J)**

Representative images of apoptosis in colonic tissues detected by TUNEL (×200), and western blot analysis for cleavage of caspase 3 and PARP. G: glands; LP: lamina propria. All quantitative data represent the mean ± SEM. *$P < 0.05$, **$P < 0.01$ versus serum IgG.

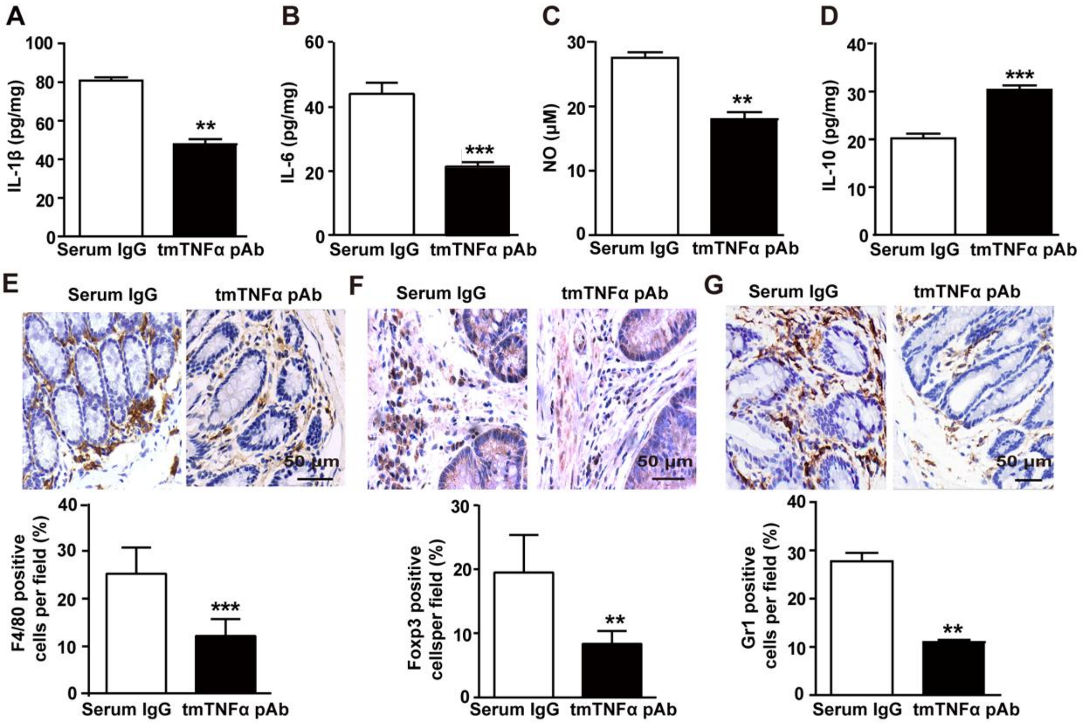

Figure S4. tmTNF-α Ab treatment in the repeated inflammation stage suppresses inflammation. tmTNF-α pAb was administered at the repeated inflammation stage as described in Figure S3A (n = 5 each group). Concentrations of IL-1β **(A)**, IL-6 **(B)**, and IL-10 **(D)** in colonic tissue homogenates were detected by ELISA. The levels of NO **(C)** in colonic tissue homogenates were measured by Griess method. **(E-G)** Representative immunohistochemistry images of F4/80$^+$ Macrophages, Foxp3$^+$Tregs and Gr1$^+$MDSCs in colonic tissues (×400) and their quantitative data. All quantitative data are presented as means ± SEM. **P < 0.01, ***P < 0.001 versus serum IgG.

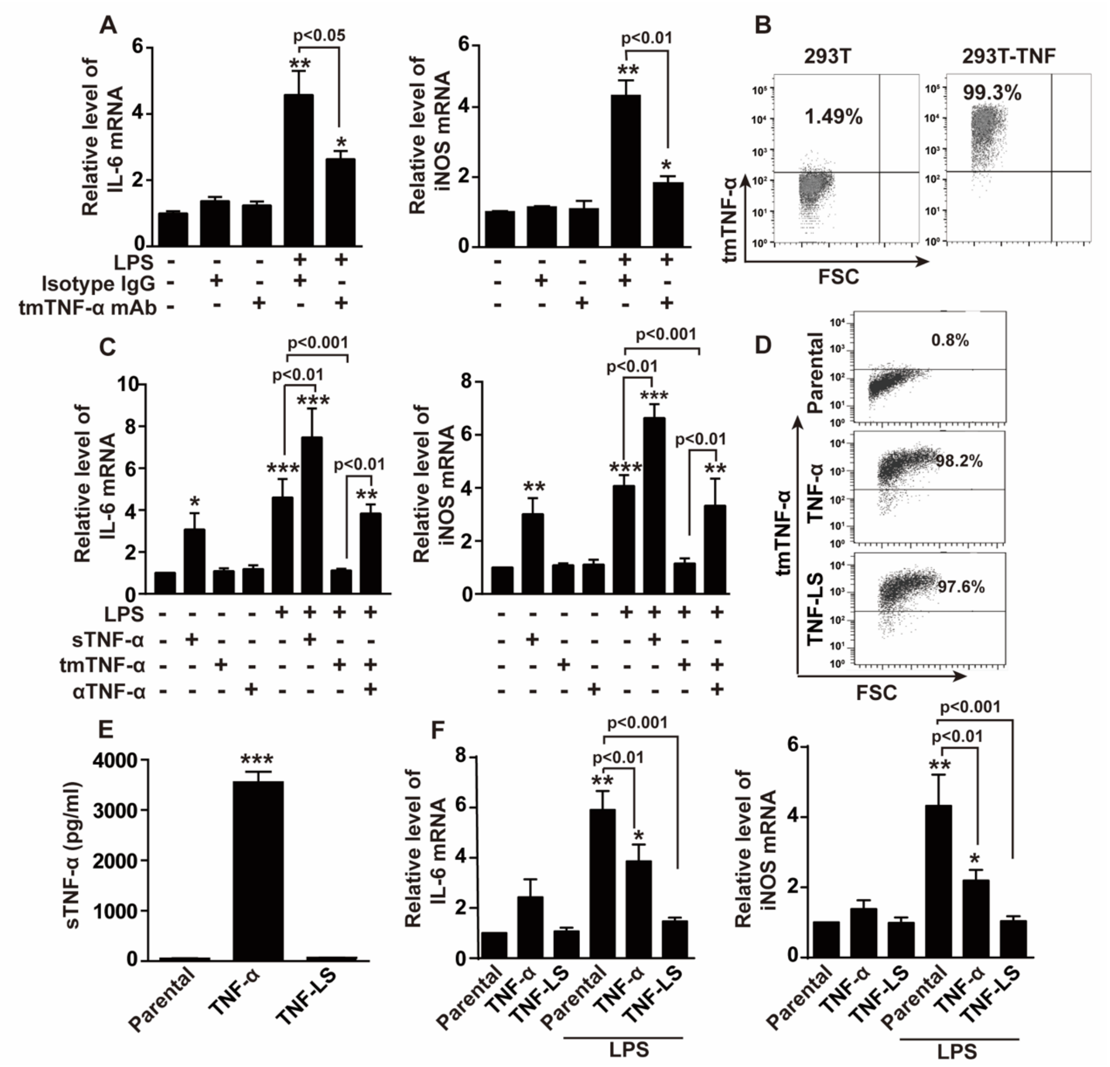

Figure S5. tmTNF-α actively suppresses LPS-induced transcription of *IL-6* mRNA and *iNOS* mRNA via dual signaling. **(A)** NCM460 cells were stimulated with 10 ng/ml LPS combined with 2 μg/ml of tmTNF-α mAb for 6 h. Isotype IgG served as a control. Relative mRNA levels of *IL-6* and *iNOS* were assessed by real-time PCR. **(B)** tmTNF-α expression on the cell surface of 293T cells stably transfected with human TNF-α cDNA detected by flow cytometry. **(C)** NCM460 cells were cocultured with 100 ng/ml sTNF-α or tmTNF-α expressed on fixed 293T cells at a ratio of 1:10 in the presence of 10 ng/ml LPS for 6 h. Relative mRNA levels of *IL-6* and *iNOS* were assessed by real-time PCR. NCM460 cells stably transfected with wtTNF-α and

TNF-LS were detected for tmTNF-α expression on the cell surface by flow cytometry **(D),** and sTNF-α levels in culture supernatants of the transfectants measured by ELISA **(E)**. **(F)** The transfectants and their parental cells were stimulated with 10 ng/ml LPS for 6 h. Relative mRNA levels of *IL-6* and *iNOS* were assessed by real-time PCR. All quantitative data are presented as means ± SEM of four independent experiments. *P < 0.05, **P < 0.01, ***P < 0.001 versus control for A and C, versus parental for E and F.

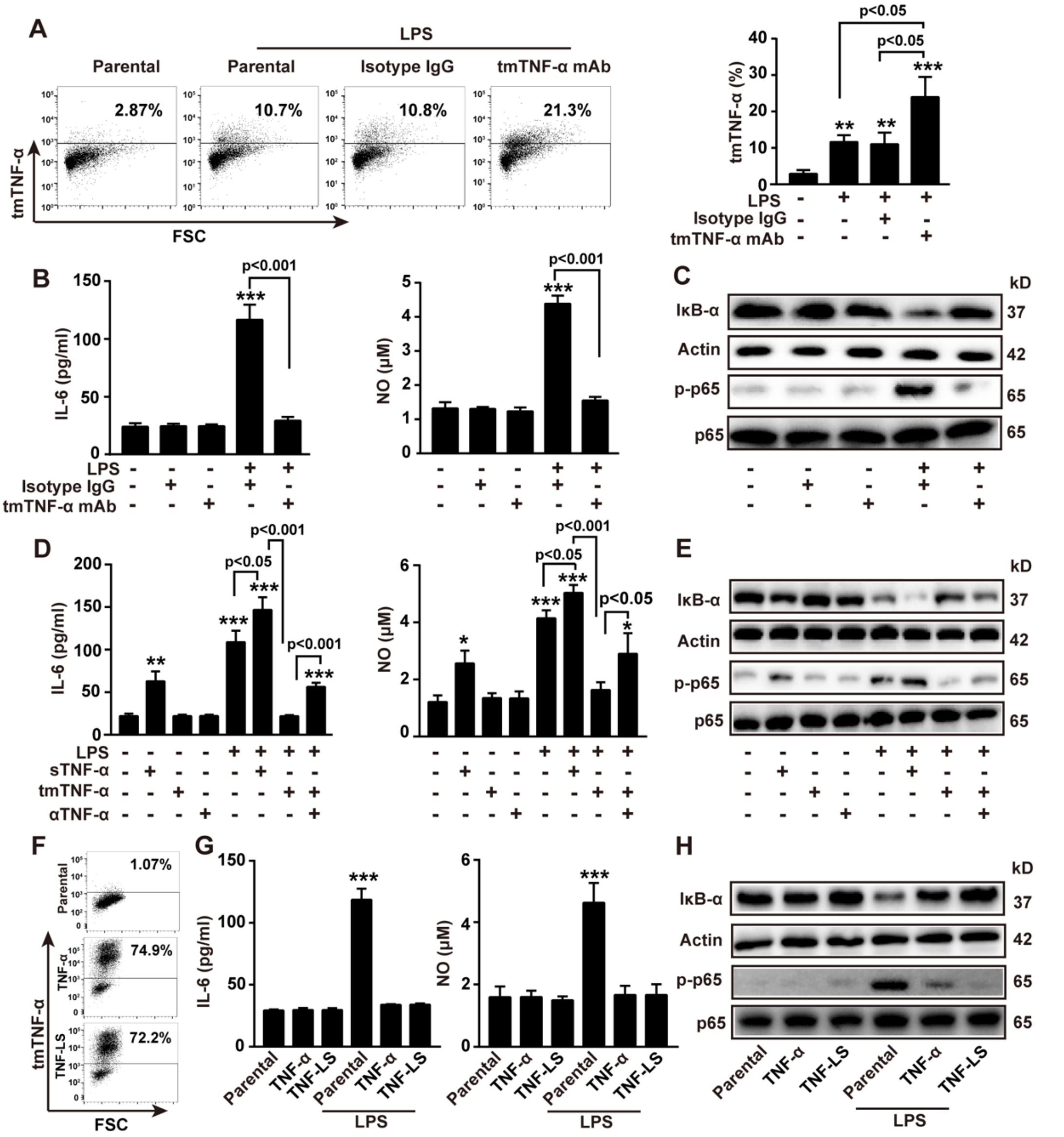

Figure S6. tmTNF-α actively suppresses the LPS-response in HCoEpiC cells. HCoEpiC cells were stimulated with 100 ng/ml LPS and 2 μg/ml tmTNF-α mAb for 6 h. Isotype IgG served as a control. **(A)** tmTNF-α expression on the cell surface assessed by flow cytometry and quantitative data. **(B)** Concentrations of IL-6 and NO in culture supernatants at 10 h after stimulation detected by ELISA. **(C)** Representative western blot of three independent experiments for IκBα degradation and p65 phosphorylation at 1 h after stimulation. **(D, E)** HCoEpiC cells were cocultured with 100 ng/ml sTNF-α or tmTNF-α stably expressed on the cell surface of fixed 293T cells at a ratio of 1:10 in the

presence of 100 ng/ml LPS. Concentrations of IL-6 and NO in supernatants at 10 h **(D)**, and representative western blot of three independent experiments for the NF-κB pathway at 1 h after stimulation **(E)**. **(F-H)** HCoEpiC cells were transfected with TNF-α and TNF-LS for 48 h and expression of the molecules on the cell surface detected by flow cytometry **(F)**. The transfectants were stimulated with 100 ng/ml LPS. Levels of IL-6 and NO in supernatants at 10 h **(G)**, and representative western blot of three independent experiments for the NF-κB pathway at 1 h after LPS stimulation **(H)**. All quantitative data represent means ± SEM of three independent experiments. *P < 0.05, **P < 0.01, ***P < 0.001 versus control for A, B, D; versus parental for G.
